# Identification of Minor Secondary Metabolites from the Latex of *Croton lechleri* (Muell-Arg) and Evaluation of Their Antioxidant Activity

**DOI:** 10.3390/molecules13061219

**Published:** 2008-06-01

**Authors:** Simona De Marino, Fulvio Gala, Franco Zollo, Sara Vitalini, Gelsomina Fico, Francesco Visioli, Maria Iorizzi

**Affiliations:** 1 Dipartimento di Chimica delle Sostanze Naturali, Università degli Studi di Napoli “Federico II”,Via D. Montesano 49, I-80131 Napoli, Italy; E-mail: sidemari@unina.it, fgala@unina.it, fzollo@unina.it; 2 Dipartimento di Biologia, Università degli Studi di Milano, Via Celoria 26, I-20133 Milano, Italy; E-mail: gelsomina.fico@unimi.it, sara.vitalini@unimi.it; 3 UMR7079,UPMC Univ Paris 06, Paris, France; E-mail: francesco.visioli@snv.jussieu.fr; 4 Dipartimento di Scienze e Tecnologie per l’Ambiente e il Territorio, Università degli Studi del Molise, Contrada Fonte Lappone, I-86090 Pesche (Isernia), Italy

**Keywords:** *Croton lechleri*, Sangre de drago, Euphorbiaceae, Latex, Secondary metabolites, Antioxidant activity

## Abstract

Dragon’s blood (*Sangre de drago*), a viscous red sap derived from *Croton lechleri* Muell-Arg (Euphorbiaceae), is extensively used by indigenous cultures of the Amazonian basin for its wound healing properties. The aim of this study was to identify the minor secondary metabolites and test the antioxidant activity of this sustance. A bio-guided fractionation of the *n*-hexane, chloroform, *n*-butanol, and aqueous extracts led to the isolation of 15 compounds: three megastigmanes, four flavan-3-ols, three phenylpropanoids, three lignans, a clerodane, and the alkaloid taspine. In addition to these known molecules, six compounds were isolated and identified for the first time in the latex: blumenol B, blumenol C, 4,5-dihydroblumenol A, *erythro*-guaiacyl-glyceryl-β-*O*-4’-dihydroconiferyl ether, 2-[4-(3-hydroxypropyl)-2-methoxyphenoxy]-propane-1,3-diol and floribundic acid glucoside. Combinations of spectroscopic methods (^1^H-, ^13^C- NMR and 2D-NMR experiments), ESI-MS, and literature comparisons were used for compound identification. *In vitro* antioxidant activities were assessed by DPPH, total antioxidant capacity and lipid peroxidation assays. Flavan-3-ols derivatives (as major phenolic compounds in the latex) exhibited the highest antioxidant activity.

## Introduction

*Croton lechleri* (Muell-Arg) (Euphorbiaceae), is a tree which grows in the low mountainous areas of the Peruvian Andean regions, as well as in Colombia, Ecuador and Bolivia and it is known for its therapeutic properties. The bark, when slashed, releases a reddish or yellowish latex called “sangre de drago” or “sangre de grado” or “dragon’s blood”. The blood-red latex or sap is a common household remedy in Peru and in other Latin American countries, where indigenous tribes use it internally and externally to stop bleeding, help heal wounds, and treat intestinal ailments [1]. The results of *in vitro* and *in vivo* studies support the use the viscous latex, which exhibits antioxidant [2,3] antiviral [4] and anti-inflammatory [5] activities, in addition to being efficacious in the treatment of different types of diarrhoea, including cholera [6]. The oral administration of a compound, termed SP-303, isolated from the bark latex by Ubillas *et al*. [4], leads to positive results in the treatment of traveller’s diarrhoeas [7] and diarrhoeal episodes in AIDS patients [8]. Recently a novel extract, named SB-300, was formulated and made commercially available [9]. When applied to the skin for treating abrasions and blisters, the red sap forms a seal, protecting the lesion [10,11]. It is applied topically to reduce the symptoms of insect bites with a reduction of swelling and redness [10,12]. The sap has been used in the treatment of several types of tumors [13,14,15]. Since free radicals may participate in the early stages of carcinogenesis, recently antioxidant activity was evaluated against the oxidative damages induced by apomorfine in *Saccaromices cerevisiae* [16].

The chemical constituents of several species of genus *Croton* have been extensively investigated. The characteristic secondary metabolites are proanthocyanidins, which account for up to 90% of dry weight and many polyphenolic components such as catechin, epicatechin, gallocatechin, epigallocatechin and dimeric procyanidins B-1 and B-4 [17]. Several minor constituents were also identified: clerodane diterpenoids such as korberin A and B [18], bincatriol, crolechinol, crolechinic acid [19] and the dihydrobenzofuran lignan 3’,4-*O*-dimethylcedrusin [5]. Work on *C. lechleri* led to the isolation of a benzylisoquinoline-like alkaloid taspine in the sap [1,11] and thaliporphine and glaucine in the leaves [14]. Taspine and the lignan 3’,4-*O*-dimethylcedrusin are thought to be responsible for the wound healing actions of *sangre de drago*, because of their stimulatory actions on wound repair [2,11,15]. 

## Results and Discussion

In the present study we have identified minor chemical components of *Croton* sap to support its medicinal exploitation. The raw material was subjected to a bio-guided fractionation process to isolate and identify the main antioxidant non-volatile components. Over the past few years, considerable effort has been devoted to optimizing the extraction of phenolic compounds from herbs and plants. We show here that the crude extracts of *C. lechleri* sap contains a great variety of phenolic components, along with several compounds of very different chemical structures. Our systematic study established a correlation between the chemical composition of the phenolic fractions and their antioxidant activities. Chromatographic separation by Sephadex^®^ LH-20 was followed by DCCC (droplet counter-current chromatography), which is a key step in the purification of complex mixtures of natural compounds. The final purification by HPLC led to the identification of blumenol B (**1**), blumenol C (**2**) [20], 4,5-dihydroblumenol A (**3**) [21], (–)-epicatechin (**7**), (+)-catechin (**8**), (–)-epigallocatechin (**9**) and (+)-gallocatechin (**10**) [17], 3’,4-O-dimethylcedrusin (**4**) [5], (±) *erythro*-guaiacyl-glycerol-β-*O*-4’-dihydroconiferyl ether (**5**) [22], 2-[4-(3-hydroxypropyl)-2-methoxyphenoxy]-propane-1,3-diol (**6**) [23], floribundic acid glucoside (**14**) [24] and taspine (**15**) [5].

**Figure 1:**
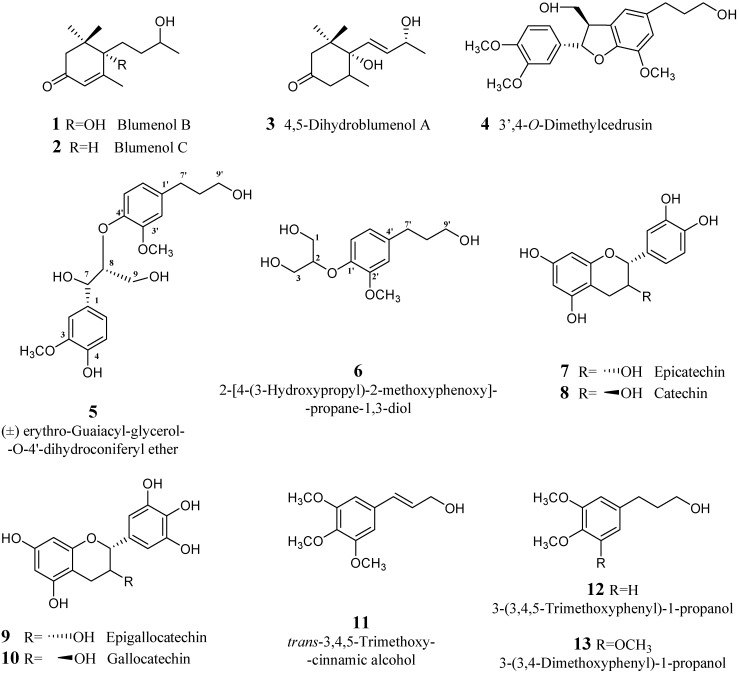

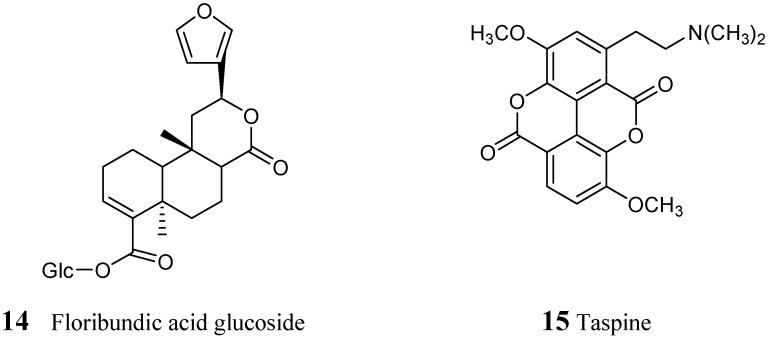
Secondary metabolites isolated from the latex of *Croton lechleri* (Muell-Arg).

Among the 15 secondary products isolated in the present study (Figure 1), the nor-isoprenoid derivatives blumenol B (**1**), blumenol C (**2**), 4,5-dihydroblumenol A (**3**), the lignane derivatives **5** and **6** and the clerodane glucoside **14** have not previously been isolated from the genus *Croton.* Their structures were confirmed by comparison of the ^1^H- and ^13^C-NMR and mass spectra data with published values. 

Molecules with megastigmane skeletons, albeit having been described, are relatively rare in the *Euphorbiaceae* family. The existence of proanthocyanidins seems to be the characteristic of *C. lechleri*, however we did not identify any oligomeric components, while the most abundant compounds isolated were (–)-epicatechin (**7**), (+)-catechin (**8**), (–)-epigallocatechin (**9**) and (+)-gallocatechin (**10**), previously found in several *Croton* saps.

The distribution of phenolic flavan-3-ols between the *n*-butanol and the CHCl_3_ extracts revealed some interesting features. The principal component in the *n*-butanol extract was gallocatechin (**10**; 11.8 mg), followed by epigallocatechin (**9**; 5.2 mg) and epicatechin (7; 3.0 mg). Epicatechin has also been isolated, in lower concentrations, as a component of the CHCl_3_ extract, along with catechin (**8**; 4.0 mg); reflecting these compositions, the highest antioxidant activity was detected in the *n*-butanol extract, compared to the CHCl_3_ extract. In the chloroformic extract, flavan-3-ol components were accompanied by several other phenolic constituents.

Lignans were reported in the latex of various *Croton* sp. [5]. Along with the known 3’,4-*O*-dimethylcedrusin (**4**), two rare components were identified, for the first time, in the title species: 1-(4-hydroxy-3-methoxyphenyl)-2-[4-(3-hydroxypropyl)-2-methoxyphenoxy]-propane-1,3-diol (**5**) and 2-[4-(3-hydroxypropyl)-2-methoxyphenoxy]-propane-1,3-diol. (**6**). 

Compound **5**, also termed *erythro*-guaiacyl-glycerol-β-*O*-4’-dihydroconiferyl ether, was identified on the basis of its spectroscopic data. The EI-MS spectrum showed a molecular ion peak at *m/z* 378 [M]^+^ which is consistent with a molecular formula of C_20_H_26_O_7._

The ^1^H-NMR (Table 1) and ^1^H-^1^H COSY spectra revealed four spin systems. Six aromatic protons were attributed to two aromatic rings: the first one was 1,3,4-trisubstituted, with a narrow doublet at δ 7.02 (*J*= 1.6 Hz), a doublet at δ 6.77 (*J*= 8.1 Hz) and at δ 6.87 (dd, *J*=1.6, 8.1 Hz). The second aromatic system was 1’,3’,4’-trisubstituted and characterized by signals at δ 6.98 (d, *J*= 8.1 Hz), δ 6.73 (dd, *J*= 8.1, 1.7 Hz) and δ 6.87 (d,*J*= 1.7 Hz). The COSY spectrum also indicated the presence of a glycerol-type moiety from C-7 to C-9 [δ 4.88 (d, *J*=4.7 Hz), δ 4.20 (ddd, *J*= 4.7, 3.8, 3.0 Hz) and δ 3.47 (dd, *J*=12.1, 3.8 Hz), δ 3.72 (dd, *J*=12.1, 3.0 Hz)]. In accordance with the *erythro* relative configuration, a coupling constant value of 4.7 Hz between H-7 and H-8, was observed [25,26]. 

**Table 1:** ^1^H- and ^13^C-NMR data (CD_3_OD, 500 and 125 MHz) of compounds **5** and **6**.

	5		6		
Position	δ_H_	δ_C_	Position	δ_H_	δ_C_
1	-	132.7	1	3.75 d (*J*= 5.1 Hz)	61.7
2	7.02 br s (*J*= 1.6 Hz)	118.6	2	4.15 q	83.0
3	-	147.7	3	3.75 d (*J*= 5.1 Hz)	61.7
4	-	146.2	1’	-	146.5
5	6.77 d (*J*= 8.1 Hz)	115.6	2’	-	151.6
6	6.87 dd (*J*= 8.1, 1.6 Hz)	119.5	3’	6.86 d (*J*=1.5 Hz)	113.8
7	4.88 d (*J*= 4.7 Hz)	73.2	4’	-	138.1
8	4.20 ddd (*J*= 4.7, 3.8, 3.0 Hz)	87.0	5’	6.74 dd (*J*= 8.1, 1.5 Hz)	121.6
9	3.72 dd (*J*= 12.1, 3.0 Hz) 3.47 dd (*J*= 12.1, 3.8 Hz)	60.7	6’	6.99 d (*J*= 8.1 Hz)	119.2
1’	-	137.2	7’	2.62 t (*J*= 8.1 Hz)	32.4
2’	6.87 d (*J*= 1.7 Hz)	112.8	8’	1.82 m	35.3
3’	-	150.5	9’	3.56 t (*J*= 6.6 Hz)	61.9
4’	-	146.4	-OCH_3_	3.84 s	56.1
5’	6.98 d (*J*= 8.1 Hz)	110.5			
6’	6.73 dd (*J*= 8.1, 1.7 Hz)	120.9			
7’	2.63 t (*J*= 8.0 Hz)	31.8			
8’	1.82 m	34.6			
9’	3.55 t (*J*= 6.4 Hz)	61.0			
-OCH_3 _in 3	3.83 s	55.1			
-OCH_3 _in 3’	3.85 s	55.3			

Two methoxyl groups (δ 3.83 and δ 3.85) and a C-3 chain were also detected in the ^1^H-NMR spectrum. An HSQC experiment allowed the assignment of protons to the corresponding carbons while the connectivity networks through the moiety, were detected by an HMBC experiment. The most important HMBC correlation peaks were observed between H-6’ (δ 6.73) and C-7' (31.8 ppm), H-8 and C-1 (132.7 ppm), H-6 (δ 6.87) and C-7 (73.2 ppm). Further information was obtained by a ROESY experiment, which showed dipolar correlations between –OCH_3_ (δ 3.83) and H-2 (δ 7.02) and –OCH_3_ (δ 3.85) and H_2_-7’ (δ 2.63). Therefore the structure of 5 was proposed as 1-(4-hydroxy-3-methoxyphenyl)-2-[4-(3-hydroxypropyl)-2-methoxyphenoxy]-propane-1,3-diol, also known with the trivial name *erythro*-guaiacyl-glycerol-β-*O*-4’-dihydroconiferyl ether. In the literature, this compound has only been previously reported following isolation from *Pinus silvestris* [22].

The molecular formula of compound **6** was deduced as C_13_H_20_O_5 _from EI-MS, which showed a [M]^+^ peak at *m/z* 256. The ^1^H-NMR (Table 1) and ^1^H-^1^H COSY spectra showed two one-proton doublets at δ_Η_ 6.99 (*J*=8.1Hz) and δ_Η_ 6.86 (*J*=1.5 Hz) and a one-proton double doublet at δ_Η_ 6.74 (*J*=8.1, 1.5 Hz), suggesting a typical 1,2,4-trisubstituted aromatic ring. These spectra also included a singlet at δ 3.84, ascribable to a methoxyl function, a sequence of three methylene groups (δ 2.62, δ 1.82 and δ 3.56), indicating a propanol side chain and a quintuplet at δ 4.15 (H-2) coupled with four protons resonating at δ 3.75 (H_2_-1 and H_2_-3). The structure of **6** and the complete assignment of its ^1^H- and ^13^C-NMR data was accomplished by HSQC and HMBC experiments. The key correlations in the HMBC allowed the arrangement of the three substituent groups on the aromatic ring. The correlation between H-2 (δ 4.15) and C-1’ (146.5 ppm) revealed that the glycerol moiety was linked to C-1’; the correlation of H-3’ (δ 6.86) and C-2’ (151.6 ppm) showed a 2-OMe position; the correlation of H-3’ (δ 6.86) with C-7’ (32.4 ppm) established the location of propanol side chain in C-4’. Most relevant ROESY connectivity were observed between –OCH_3_ (δ 3.84) and H-3’ and H_2_-1 (δ 3.75) with H-6’ (δ 6.99). Thus the structure of **6** was defined as 2-[4-(3-hydroxypropyl)-2-methoxyphenoxy]-propane-1,3-diol. This compound was previously identified in wine from *Vitis vinifera* and has been described as the corresponding peracetylated derivative [23].

Several clerodane diterpenoids have been identified in previous phytochemical studies [18], but until now, floribundic acid glucoside (**14**) had never been detected in any species of the genus *Croton*. 

### Antioxidant activity.

All crude extracts (*n*-hexane, CHCl_3_, *n*-BuOH and aqueous residue) obtained from the *C. lechleri* latex were analyzed for their phenolic content. The highest concentration of phenols (306 mg/g) was found in the *n*-BuOH extract and the lowest one in the *n*-hexane extract (4.84 mg/g). Consequently, the latter was not further investigated. The CHCl_3_, *n*-BuOH and aqueous residues were tested to evaluate their antioxidant potential (Table 2). 

**Table 2:** Phenolic content and antioxidant activity of extracts, fractions, and pure compounds isolated from *C. lechleri* latex.

Sample	Phenols (mg/g extract)	Antioxidant capacity (mEq uric acid)		DPPH (IC_50_)
		1 μM	10 μM	μM
Ascorbic acid	n.a	0.36±0.02	0.33±0.02	9.65 μM
Trolox^®^	n.a	0.85±0.07	1.77±0.18	12.9 μM
Quercetin	n.a	0.75±0.06	2.17±0.17	4.37 μM
*n*-Hexane extract	4.84	/	/	/
CHCl_3_ extract	41.87	0.09±0.01	0.15±0.01	0.09 μM
*n*-BuOH extract	306.01	0.10±0.01	0.65±0.05	0.875 μM
H_2_O residue extract	34.37	0.05±0.01	0.12±0.01	12.4 μM
Fraction 1	212.90	0.01±0.01	0.83±0.07	13.1 μM
Fraction 2	314.55	0.02±0.01	1.81±0.14	6.10 μM
Fraction 3	546.47	0.01±0.01	1.11±0.08	10.9 μM
Fraction 4	330.51	0.03±0.01	0.66±0.05	1.41 μM
Gallocatechin	n.a	0.16±0.03	1.35±0.10	10.0 μM
Epigallocatechin	n.a	0.39±0.05	2.05±0.11	0.561 μM
Epicatechin	n.a	0.09±0.01	0.36±0.03	19.3 μM

Experiments were performed in triplicate; results are mean ± SD.

As expected, the *n*-BuOH extract showed the highest activity; in particular it exhibited a scavenging action toward the stable radical DPPH which was stronger than that of all three reference compounds, i.e. quercetin, Trolox^®^, and ascorbic acid. Only the Sephadex^®^ fractions (1, 2, 3 and 4) obtained from this extract were examined for their antioxidant activity (Table 2). The scavenging abilities of the four fractions were comparable with those of the standards; the overall antioxidant capacity yielded similar results, but only at 10 μM concentrations. In both tests, fractions 2 and 3 showed the strongest activity. From these fractions, epigallocatechin and gallocatechin were isolated and then investigated. Two pure compounds exhibited remarkable antioxidant activities (Table 2) both in terms of DPPH removal (with values lower than those of quercetin and Trolox^®^) and of antioxidant capacity (with results similar to those of the quercetin and Trolox^®^). Epicatechin was isolated from fraction 4; the activity of this molecule (IC_50_: 19.3 μM; Table 2) was in line with that reported in the literature [3]. The presence of epicatechin and catechin in only trace amounts in the CHCl_3_ extracts explains their lack of antioxidant activity. 

## Conclusions

In summary, we have revealed for the first time the presence in *C. lechleri* latex, of minor secondary metabolites as megastigmane, lignan, and clerodane derivatives. The results obtained by testing the *n*-BuOH extract may be ascribable to flavan-3-ols, that are the strongest antioxidants among latex phenols. The complexity of the chemical profile suggested that the role of each individual compound in the latex is important in the interpretation of the pharmacological effects exhibited by s*angre de drago*, which deserve further investigations.

## Experimental

### General

^1^H- and ^13^C-NMR spectra were determined at 500.13 and 125.77 MHz, respectively, on a Varian Unity INOVA spectrometer equipped with an indirect detection probe. Chemical shifts were referenced to the solvent signals of deuterated methanol (CD_3_OD), residual CHD_2_OD: δ_H_ 3.31, δ_C_ 49.0. Electron ionization mass spectrometry (EI-MS) and ESI-MS were recorded on a Fisons VG Prospec instrument. Droplet counter-current chromatography (DCCC) was performed on a DCC-A apparatus (Tokyo Rikakikai Co., Tokyo-Japan) equipped with 250 glass-columns. HPLC was performed using a Waters 510 pump equipped with a Waters U6K injector and a Waters 401 differential refractometer as detector, using a 30 cm x 3.9 mm; i.d., C_18_ μ−Bondapak (Waters, Milford, MA, USA) columns; flow rate was 1 mLmin^–1^. The secondary metabolites were identified by a combination of spectroscopic methods (^1^H, ^13^C NMR and 2D-NMR experiments), ESI-MS and comparison with the literature data. 

### Plant Material

The sap of *C. lechleri* (Euphorbiaceae) was collected in 2006 from the tropical region of Upper Huallaga Valley (Tingo Maria, Peru) and kept at 4 °C in the dark. A 100 mL sample of the reddish latex is deposited (N° SD-518) in the herbarium of the University of Molise (Pesche, Isernia, Italy).

### Extraction and Isolation

A small portion (150 mL) of sap was dissolved in MeOH-H_2_O (9:1, v/v, 200 mL) and extracted with *n*-hexane (3 x 150 mL), following Kupckan’s partitioning method [27]. The water content (% v/v) of the MeOH was adjusted to 40% and the resulting solution was partitioned against CHCl_3_. The MeOH was removed from the aqueous phase, which was then extracted with *n*-BuOH. This gave three extracts: *n*-hexane (80.3 mg), CHCl_3_ (1.2 g), *n*-BuOH (3.6 g) and an aqueous residue (7.8 g). The *n*-BuOH residue was chromatographed on Sephadex^®^ LH-20 (MeOH eluent). Fractions were collected and combined according to TLC analysis into four main fractions. Fraction 1 was submitted to DCCC with *n*-BuOH-Me_2_CO-H_2_O (3:1:5) in the descending mode (the upper phase was the stationary phase), to give four main fractions, which were analyzed by silica gel TLC [*n*-BuOH-HOAc-H_2_O (12:3:5) and CHCl_3_-MeOH-H_2_O (80:18:2) as eluents] and purified by HPLC with MeOH-H_2_O (15:85) as the mobile phase. Fractions 2, 3, and 4 contained mainly **7** (5.2 mg), **10** (11,8 mg) and **9** (3.0 mg), respectively. The CHCl_3 _extract was chromatographed by DCCC using CHCl_3_-MeOH-H_2_O (7:13:8) in ascending mode (the lower phase was the stationary phase). Six fractions (A-F) were obtained and purified by HPLC as follows. Fraction A was purified with MeOH-H_2_O (1:9) and contained **7** (2.9 mg) and **8** (4 mg); fraction B, eluted with MeOH-H_2_O (15:85), contained **6** (1.6 mg); fraction C contained mainly **14** (1.2 mg) and was eluted with MeOH-H_2_O (4:6); fraction D, eluted with MeOH-H_2_O (2:8), contained **3** (9.5 mg), **2** (2.2 mg), **12** (0.8 mg), and **5** (1.3 mg); Compounds **4** (3.9 mg), **11** (2.8 mg) and **13** (1.7 mg) were obtained from fraction E, eluted with MeOH-H_2_O (25:75); fraction F, eluted with MeOH-H_2_O (3:7), contained mainly **1** (8.6 mg).

### Isolation and identification of alkaloids

*C. lechleri* latex (50 mL) was lyophilized to yield 7.6 g of reddish-powdered latex. A portion (3.0 g) of the powdered latex was combined with distilled H_2_O (150 mL) and acidified with concentrated HCl. The aqueous layer was continuously extracted with CHCl_3 _for 12 h and its pH was then adjusted to 8 with concentrated NH_4_OH. This fraction was then continuously extracted with CHCl_3 _over 12 h to yield the first basic fraction. Following adjustment of pH to 10 with NH_4_OH, the aqueous layer was extracted for an additional 12 h with CHCl_3 _to yield the second basic fraction. TLC examination of the two basic fractions with Dragendorff’s reagent showed reagent-positive component in the second basic fraction. This fraction was dissolved in MeOH with warming, and 15 (11.3 mg) was obtained as a white precipitate upon refrigeration.

*EI-MS data: Blumenol B* (**1**): *m/z* 224 [M]^+^ (C_13_H_20_O_3_); *Blumenol C* (**2**): *m/z* 208 [M]^+ ^(C_13_H_20_O_2_); *4,5-Dihydroblumenol A* (**3**): *m/z* 226 [M]^+ ^(C_13_H_22_O_3_); *3’,4-O-Dimethylcedrusin* (**4**): *m/z* 374 [M]^+ ^(C_21_H_26_O_6_); *(–)-Epicatechin* (**7**): *m/z* 290 [M]^+ ^(C_15_H_14_O_6_); *(+)-Catechin* (**8**): *m/z* 290 [M]^+^ (C_15_H_14_O_6_); *(–)-Epigallocatechin* (**9**): *m/z* 306 [M]^+ ^(C_15_H_14_O_7_); *(+)-Gallocatechin* (**10**): *m/z* 306 [M]^+ ^(C_15_H_14_O_7_); *Compound* **11**: *m/z* 224 [M]^+^ (C_12_H_16_O_4_); *Compound* **12**: *m/z* 196 [M]^+ ^(C_11_H_16_O_3_); *Compound* **13**: *m/z* 226 [M]^+ ^(C_12_H_18_O_4_); *Floribundic acid* *glucoside* (**14**): *m/z* 529 [M+Na]^+ ^(C_26_H_34_O_10_); *Taspine* (**15**): *m/z* 369 [M]^+ ^(C_20_H_19_NO_6_).

*(±) erythro-Guaiacyl-glycerol-β-O-4’-dihydroconiferyl ether* (**5**): 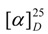 = 0° (MeOH, c 0.09); EI-MS *m/z* 378 [M]^+^ (C_20_H_26_O_7_); ^1^H- and ^13^C-NMR see Table 1.

*2*-[4-(3-Hydroxypropyl)-2-methoxyphenoxy]*-propane-1,3-diol* (**6**): EI-MS *m/z* 256 [M]^+^ (C_13_H_20_O_5_); ^1^H- and ^13^C-NMR see Table 1.

### Determination of phenolic content

Total phenols of the extracts and fractions were quantified by the Folin-Ciocalteau spectrophotometric assay, using gallic acid as the reference standard [28]. Therefore, molarity refers is expressed as gallic acid equivalents. 

### Total antioxidant capacity

The total antioxidant capacity of the samples at concentrations of 10 and 1 μM was determined by a validated method based upon the copper reduction (Cu^2+^ to Cu^+^). (BIOXYTECH^®^ AOP-490™, Oxis Research™, Portland, OR) [29]. The reference compound was uric acid and then results are expressed as mEq uric acid.

### DPPH scavenging test

Suitable aliquots of a methanolic solution containing each sample, at increasing concentrations from 1 μM to 100 μM, were added to 15 μM ethanol solution of 2,2-diphenyl-2-picrylhydrazyl radical (DPPH). Absorbance was read at 517 nm after 15 min of incubation in the dark [30]. The IC_50_ was calculated by employing Prism^®^ 4 (GraphPad Software Inc.). 
